# Effect of active external rewarming on esophageal temperature in simulated prehospital accidental hypothermia: a randomized crossover trial

**DOI:** 10.1186/s13049-025-01528-7

**Published:** 2025-12-12

**Authors:** Sigurd Mydske, Ane Marthe Helland, Nicola Borasio, Guttorm Brattebø, Øyvind Østerås, Øystein Wiggen, Jörg Aßmus, Giacomo Strapazzon, Øyvind Thomassen

**Affiliations:** 1 Department of Clinical Medicine, University of Bergen, Haukelandsveien 28, Bergen, 5009 Norway; 2 Department of Anesthesia & Intensive Care, Haukeland University Hospital, Bergen, Norway; 3 Mountain Medicine Research Group, The Norwegian Air Ambulance Foundation, Bergen, Norway; 4 Institute of Mountain Emergency Medicine, Eurac Research, Bolzano, Italy; 5 Sports and Exercise Medicine Division, Department of Medicine, University of Padova, Padova, Italy; 6 Norwegian National Advisory Unit On Emergency Medical Communication, Haukeland University Hospital, Bergen, Norway; 7 SINTEF Digital, Health Research, Trondheim, Norway

**Keywords:** Accidental hypothermia, Prehospital, Active external rewarming, Mountain medicine, Shivering inhibition, Wilderness medicine

## Abstract

**Background:**

Prehospital rewarming is crucial to reduce mortality and improve outcomes in patients with accidental hypothermia. Although international guidelines recommend combining active external rewarming with passive rewarming, the effect of active external rewarming remains unclear. We evaluated the effects of active external rewarming compared to passive rewarming in cold-stressed, nonshivering volunteers.

**Methods:**

This randomized, crossover field study aimed to recruit 12 participants and was performed in May/June 2024 in Lom, Norway. The participants were dressed in wet clothing and placed in a − 2 °C ice tunnel for 2 h or until their esophageal temperature reached 35 °C. Endogenous thermoregulation, most importantly shivering, was suppressed using a drug protocol. After cooling, their wet clothes were removed, and they were rewarmed using either a combination of three different sources of active rewarming combined with passive rewarming (intervention) or passive rewarming only (control) while lying supine on a sleeping pad. The rewarming phase lasted for one hour. The primary outcome was the mean change in esophageal temperature analyzed using analysis of covariance. Secondary outcomes included mean skin temperature rewarming rates and subjective comfort scores.

**Results:**

Eleven participants completed the trial twice in a crossover fashion. The active rewarming group showed a mean increase in the esophageal temperature of 0.15 °C (− 0.16 °C to 0.45 °C), whereas the passive rewarming group experienced a mean decrease of − 0.05 °C (− 0.33 °C to 0.23 °C), indicating a difference in rewarming rate between the active and passive rewarming groups of 0.2 °C/h (-0.03 °C to 0.42 °C). Skin temperature rewarming rate was 1.5 °C/h higher in the active rewarming group than in the control group, although the difference was not statistically significant. The subjective comfort scores favored active rewarming.

**Conclusion:**

The results from a simulated prehospital setting indicate that active warming may increase rewarming rates in accidental hypothermia treatment. Importantly, active warming may contribute to avoid a further drop in temperature during initial phases of treatment in cold environments.

**Trial registration:**

ClinicalTrials.gov identifier: NCT06342726. Registered 26.03.2024.

**Supplementary Information:**

The online version contains supplementary material available at 10.1186/s13049-025-01528-7.

## Background

Accidental hypothermia is defined as an involuntary drop in core body temperature below 35 °C [1]. It can lead to cardiac dysrhythmias, pulmonary edema, and neurological complications [2, 3]. Coagulopathy, which is a feared complication of accidental hypothermia when present in conjunction with trauma, may occur during cold exposure even if the patient is not hypothermic [4]. Accidental hypothermia is potentially fatal, and an independent risk factor for increased mortality in patients with traumatic injuries [5–7].

Prehospital treatment of accidental hypothermia involves insulating patients from the cold, ideally with a multilayered wrapping model and active external rewarming devices [1, 8, 9]. Evidence suggests that prehospital active external rewarming is not harmful [10]. Various devices with different technologies are used for this purpose, including chemical or electrical heating blankets, hot water bottles, and forced-air warmers.

Most studies on active external rewarming devices for perioperative temperature management have limited prehospital applicability because of the lack of access to standard 220 V power. Prehospital availability of chemical and electrical blankets designed for treatment of accidental hypothermia is increasing [11].

Other trials have compared rewarming devices, but evidence of their effect on core temperature, especially when used in cold environments, remains limited. Studies that have used chemical heat packs for experimental hypothermia have reported rewarming rates of 0.2–0.9 °C/h [12, 13], whereas studies on electrical heating blankets have reported rates of 0.6–1.0 °C/h [14, 15].

Several studies have compared other active external rewarming methods, such as charcoal-burning devices and forced-air warmers, reporting rewarming rates of up to 1.5 °C/h [15–17]. While these systems may be more effective than electrical or chemical solutions, their limited portability makes them less suitable for prehospital rescue services. Some studies used shivering-inhibiting drugs in an attempt to enable a reduction in core temperature and remove confounding factors such as shivering similarly to this trial, which may have influenced rewarming rates [12, 13, 15, 16].

It is critical to reestablish normothermia, particularly for patients with concomitant conditions such as traumatic hemorrhage. Further knowledge concerning the effects of current hypothermia treatment strategies is therefore essential. The aim of this study was to evaluate the combined effects of active external rewarming devices versus passive rewarming alone on esophageal temperature in cold-stressed healthy participants. The primary outcome was mean change in esophageal temperature.

## Methods

### Study design and setting

Using a crossover design, 12 participants were recruited to undergo the scenario twice. Participants were randomized to active or passive rewarming on the first study day and assigned the opposite scenario five days later. Randomization was balanced, ensuring equal allocation of participants to the intervention and control group each day. The time schedule and the placement in the tunnel in both sessions were equal for all participants. One participant had to be removed from the study as peripheral intravenous access was not achieved, leading to 11 participants in the experiment.

The power analysis has shown that, assuming a minimal clinically relevant difference in core temperature of 0.3 °C and a standard deviation of 0.2, we needed a sample size of 9 participants in each group to achieve a power of 0.9 for a two-sided t-test at a significance level of 0.05. The power calculation was performed for a parallel design. This was a conservative choice since the crossover design has better power than the parallel design does and the ANCOVA has better power than the t-test does.

In both scenarios, participants were placed supine on a Mammut Bamse Extreme sleeping pad (Mammut Sports Group, Seon, Switzerland, R value of 1.9) while wearing soaking-wet cotton clothing. The cooling phase ended after 2 h of cooling or when the esophageal temperature reached 35 °C. At the end of the cooling phase, the wet clothing was cut off and removed using a gentle log roll into the prepared wrapping model. Wrapping completion marked the start of the 1-h rewarming phase.

The experiments were conducted in an ice tunnel within a glacier at *Klimapark2469 (*https://klimapark2469.no/en/*)* in Lom, Norway, from 28.05–02.06.2024. It is located approximately 1850 m above sea level and has a constant temperature of approximately − 2 °C and no wind.

### Inhibition of endogenous thermoregulation

The research protocol for this experiment was based on a protocol developed by Helland et al. for inhibition of endogenous thermoregulation in simulated accidental hypothermia [18]. We used an adapted version of the protocol to maximize the rewarming period. There was no additional administration of Meperidine based on subjective scores in this experiment, only predefined doses at fixed intervals for all participants. Participants received 30 mg of oral buspirone 1 h before the experiment. Before the cooling phase, they received an intravenous dose of meperidine (1 mg/kg) divided into five aliquots administered at 2 min. intervals. During the 3 h experiment, maintenance boluses of 0.5 mg/kg were administered intravenously every 25 min. 3 h (2 h cooling, 1 h rewarming) is the maximum duration of this protocol, as a longer time frame would entail exceeding the maximum recommended cumulative dose per day for Meperidine. Prophylactic ondansetron (4 mg) was administered intravenously before the first meperidine dose, with an additional 4 mg given during or after the experiment, if needed. A five-day wash-out period between sessions was selected to ensure complete washout of the medication.

### Selection of participants

We recruited participants through an open invitation. This was distributed through our network of prehospital healthcare providers, both professional and volunteer search and rescue services. Potential candidates were screened for eligibility, and a complete list of the inclusion and exclusion criteria can be found in the supplementary files.

### Active rewarming and passive rewarming scenarios

**Fig. 1:**
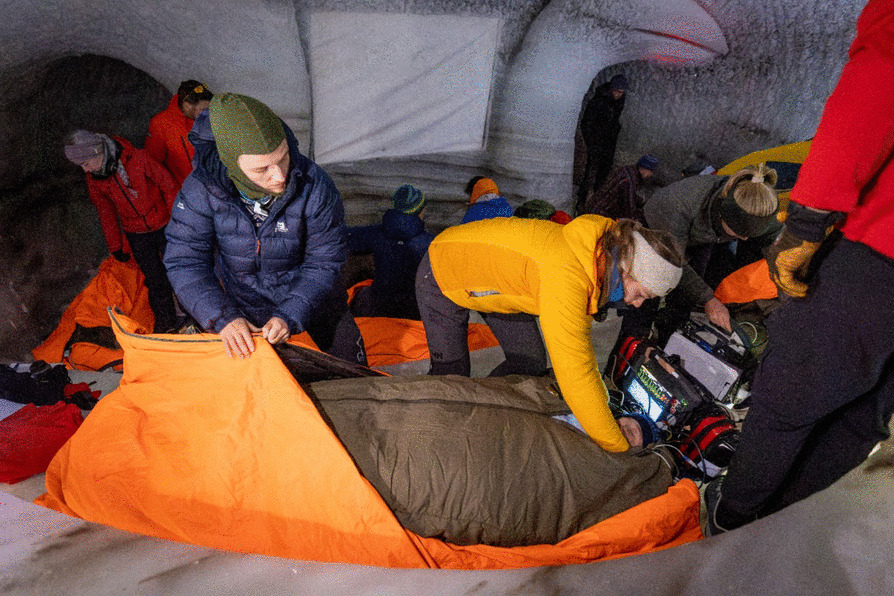
A photo from inside the ice tunnel during the wrapping phase between cooling and rewarming, showing the application of the layers in the “burrito wrap.” Photo: Rolf Magnus Sæter, The Norwegian Air Ambulance Foundation

**Fig. 2:**
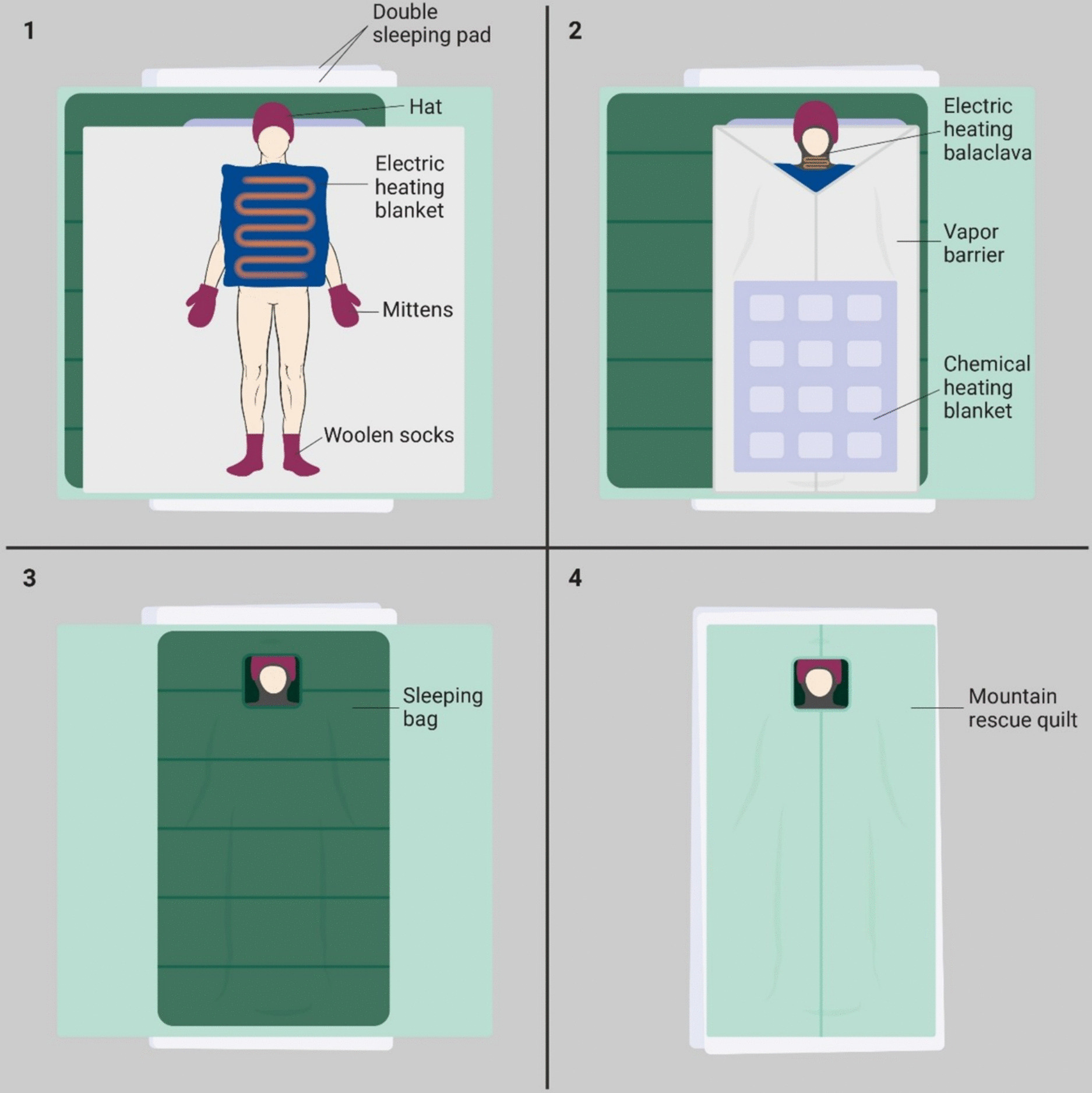
Patient wrapping in the active rewarming scenario with a multilayered model and different sources of active external rewarming. This shows the participant (1) after they have had their wet clothing removed and been placed in the wrap with the electric blanket directly on the skin of their torso, (2) the applied heated balaclava, vapor barrier and chemical heating blanket outside the barrier to avoid direct skin contact, (3) the applied sleeping bag as an insulating layer and (4) the wind- and waterproof mountain quilt as the outer layer. Figure design inspired by the “Cold card” developed by Giesbrecht [19]

The participants were wrapped in a “burrito” wrap in accordance with hypothermia management guidelines [8, 9]. The setup included a vapor barrier (ASAP JONA 200™, ASAP Norway, Skien, Norway, two sheets taped together to achieve a complete seal), an insulating winter extreme sleeping bag (Carinthia Defense 6 G-LOFT 435 g/m^2^, Goldeck Textil GmbH, Seeboden, Austria), and a windproof and waterproof mountain quilt (Jerven Extreme Primaloft 170 g/m^2^, Jerven AS, Odda, Norway) (Fig. 1). An additional insulating sleeping pad (Z-lite, Therm-a-Rest, Seattle, WA, USA, R value 2.0) was used during rewarming, providing a total R value for the sleeping pads of 3.9. In the active rewarming group, participants received additional warming devices: an electric heating blanket on the torso (PAX Warming blanket, 48 × 48 cm, X-CEN-TEK GmbH, Germany) directly on the skin as per manufacturer recommendations, a chemical heating blanket on the lower body (Ready Heat II, 86 × 122 cm, TechTrade, Orlando, FL, USA) outside the vapor barrier, and a heated balaclava (HAT Hypothermia Active Treatment, Northerm Medical, Minitech AS, Ridabu, Norway) were added (Fig. 2).

### Measurements and outcomes

We measured esophageal temperature continuously using a transnasal esophageal probe (Type ER400-9, Smiths Medical, London, UK. Accuracy ± 0.1 °C) [20] which was recorded at 1-min intervals with a Corpuls3 multimonitor (Corpuls, GS Elektromedizinische Geräte G. Stemple GmbH, Germany), which also monitored 4-lead continuous electrocardiogram (ECG), peripheral oxygen saturation (SpO_2_), and noninvasive blood pressure every 15 min.

Skin temperature was recorded every 30 s using thermistors (iButton®, Maxim Integrated Products, Inc.) placed at seven predefined locations (forehead, lower arm, hand, foot, lower leg, thigh, abdomen). Mean skin temperature was calculated using a modified weighted formula by Hardy and Dubois [21, 22]. The main reason for measuring skin temperature was to evaluate risk of frostbite during cooling or burns during rewarming. Subjective shivering and thermal comfort scores were recorded every 20 min during the cooling and rewarming phases. The questionnaire is available in the supplementary materials. Instrumentation and baseline measurements were performed before cold exposure and meperidine administration.

### Analysis

We used descriptive methods to characterize the samples. Differences in esophageal and mean skin temperatures between the rewarming techniques were assessed using analysis of covariance (ANCOVA) at each time point in the rewarming phase, i.e., the linear model of the outcome at the measured time point depending on the scenario, adjusted for the outcome at baseline and individual random intercept. The random intercept accounted for the crossover design, assuming no carryover effect. ANCOVA for esophageal temperature at the end of the rewarming phase served as the primary outcome analysis. Subjective comfort was analyzed graphically (bar plots and forest plots) and statistically using a paired *t*-test at measured time points. Note that we abstained from using an ANCOVA as best analysis because almost all measurements were constant at baseline. The significance level was set at 0.05. Since the primary outcome was only the effect at the end of the rewarming we did not adjust for multiple testing, even if we provided a test at each time point. Model assumptions were checked using residual plots. R 4.4.0 [23] was used for computation and data handling, while MATLAB 2023b (The MathWorks Inc., Natick, MA) was used for graphical presentations.

### Ethics statements

The Norwegian Regional Ethics Committee for Medical and Health Research (2024/714469) and the Data Protection Officer of Haukeland University Hospital approved this study, which was registered at ClinicalTrials.gov (NCT 06342726, reg. 26.03.2024). All participants provided written informed consent.

## Results

### Characteristics of the study subjects

Twelve volunteers were recruited and screened for eligibility. There was one case of post-randomization dropout due to the inability to obtain intravenous access. Eleven participants completed both scenarios of the trial, and all the measures and observations were complete (Table 1).

**Table 1:** Baseline characteristics of the research participants

Rewarming method in the first of two runs^1^		
	Passive	Active
	*n* = 6	*n *= 5
Age (years)^2^	24.5 [21, 54]	25 [22, 32]
Sex (Female)^3^	4 (75%)	3 (60%)
Height (cm)^2^	170 [156, 190]	173 [160, 190]
Weight (kg)^2^	74.5 [72, 82]	68 [55, 90]
BMI^2^	25.8 [20.4, 29.6]	24.9 [20.8, 26.6]

^1^Opposite scenarios completed five days later

^2^Median [min, max]

^3^N(%)

### Mean esophageal temperature

**Fig. 3:**
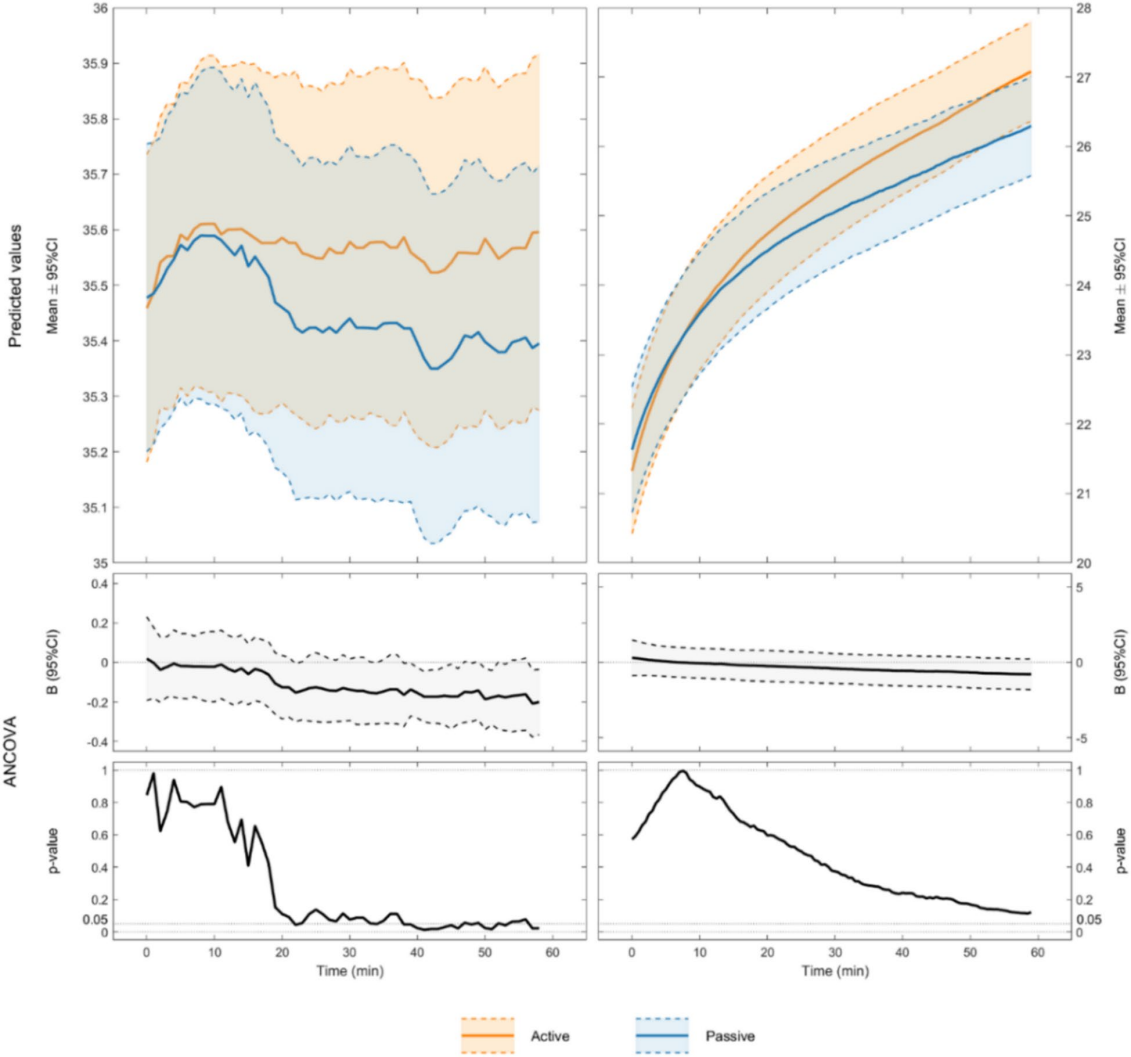
Mean esophageal (left) and skin temperatures (right) during the 1-h rewarming phase, with continuous ANCOVA calculations comparing active and passive rewarming groups. The figure shows the model-predicted average values for each group with 95% confidence intervals. “B (95% CI)” represents the estimated regression coefficient from the ANCOVA model, with 95% confidence intervals

The active external rewarming group had a mean esophageal temperature increase of 0.15 °C (− 0.16 °C to 0.45 °C) at the end of the 1-h rewarming phase, whereas the passive rewarming group experienced a mean decrease of − 0.05 °C (− 0.33 °C to 0.23 °C) (Fig. 3). This means that active external rewarming increased the esophageal temperature rewarming rate by 0.2 °C (−0.03 °C to 0.42 °C) in one hour. Both groups showed an initial temperature increase during the first 10 min. Thereafter, the mean esophageal temperature appeared to stabilize in the active rewarming group but decreased in the passive rewarming group.

### Mean skin temperature

Mean skin temperature increased in both groups during rewarming. The active rewarming group experienced an increase of approximately 7 °C, whereas the passive rewarming group experienced an increase of approximately 5.5 °C.

### Thermal comfort

**Fig. 4:**
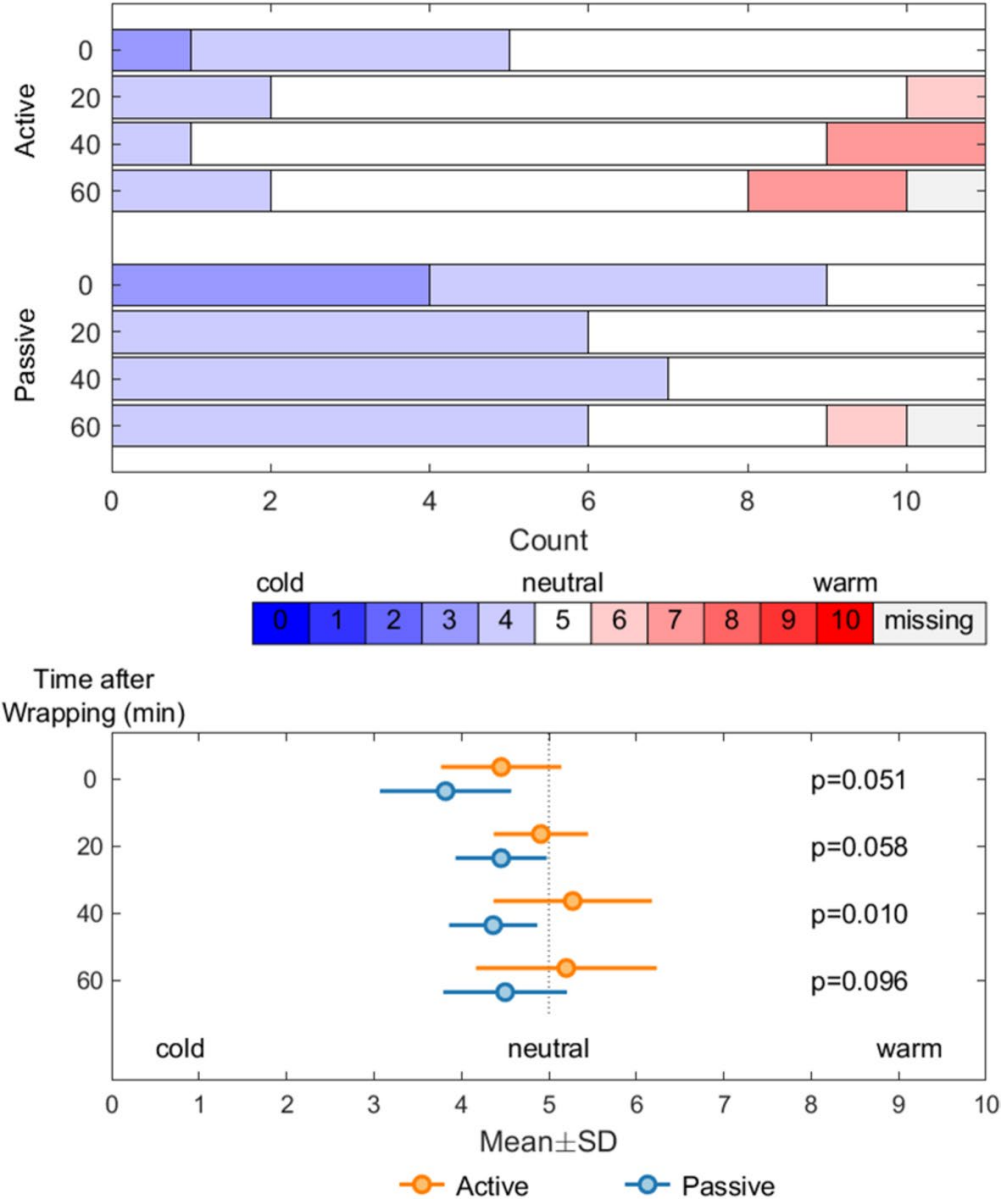
Subjective scores of thermal discomfort in the active and passive rewarming groups during the rewarming phase of the experiment. The top graph displays the distribution of responses concerning thermal comfort in the two groups as the experiments progressed, and the bottom graph displays mean values and standard deviation between the groups throughout the experiment. Two data points are missing because they were mistakenly not recorded

Subjective evaluations of comfort indicated that participants in the active rewarming group felt warmer than those in the passive rewarming group (Fig. 4), with differences increasing throughout the experiment.

### Experimental procedure

No significant changes in ECG patterns, blood pressure, or heart rate were observed. One participant in the passive rewarming group experienced an unexpectedly large drop in esophageal temperature after the first round of experiments. The esophageal temperature at exit was 35.2 °C but decreased to 33.7 °C when walking out of the ice tunnel after the end of the experiments. Although this occurred after the experiment ended, the temperature drop fell below our safety limit of 34 °C. Consequently, we increased the threshold for ending the cooling phase from 35 °C to 35.5 °C to ensure participant safety in the remaining experimental runs.

## Discussion

The main finding of this study is that nonshivering, young, healthy individuals may continue to lose heat without external warming in cold environments. Insulation and aggressive active external rewarming can help maintain or slightly increase esophageal temperature.

This study showed that even with the recommended wrapping method and multiple active rewarming devices, we only managed to achieve a difference in esophageal temperature rewarming rate of 0.2 °C/h in our nonshivering volunteer participants in a cold environment. The esophageal temperature decreased in the passive rewarming scenario, even though the participants were extensively insulated. Our observed rewarming rates were lower than those reported in similar studies [12–16], which may be attributed to variations in ambient air temperatures during rewarming, afterdrop before initiation of rewarming, or study design differences. Our study used a different protocol for inhibition of shivering than previous studies, and used cold ambient air as the cooling medium. The rewarming phase in our study was conducted in the same, cold environment as the cooling phase, similar to prehospital conditions during rescue.

All participants in this study were nonshivering, perhaps making the observed rewarming rates less applicable to clinical settings where patients are experiencing cold-induced shivering. However, many patients with hypothermia are unable to shiver, and shivering ability will decrease if core temperature drops towards Revised Swiss System Stage 2 and 3 [24]. Several factors may affect shivering ability, including exhaustion, traumatic brain injury, intoxication, or excessively low core temperatures. Iatrogenic causes such as analgesia or anesthesia may also inhibit shivering. Thus, the findings of this study are likely more applicable to patients who are not shivering. As seen in this study, nonshivering individuals will continue to lose heat in cold environments despite substantial passive insulation unless active external rewarming is applied, which may be particularly relevant during prolonged rescue efforts.

The drugs used in this trial also influence cutaneous vasomotor activity, a key thermoregulatory mechanism. The drugs may have induced vasodilation, increasing cutaneous circulation and heat uptake. The effect of active rewarming devices on a patient with vasoconstriction could be even lower due to vasoconstriction. This supports our conclusion of a modest rewarming effect.

The goal of hypothermia management is to maintain or restore the patient towards normothermia. In mild hypothermia, energy replenishment and movement to increase endogenous heat production should be considered, which is possible in some situations [25]. However, in moderate or severe stages of hypothermia, this approach is not advisable due to the high risk of arrhythmia. These patients should be handled carefully, wrapped in a hypothermia wrap, and treated with active external rewarming devices [26].

Our results suggest that current external rewarming methods and technologies may contribute to rewarming patients towards normothermia. However, the surprisingly small difference in achieved rewarming rate of 0.2 °C/h should trigger initiatives to develop more effective technologies for prehospital treatment of accidental hypothermia. Thermal transfer to participants could be improved by increasing the body surface area in contact with the heat source or by increasing the device temperature. Increased body surface area available for thermal transfer may be achieved by, for example, using a heated mattress, allowing thermal transfer to the posterior body surface.

Increasing the device temperature poses a risk of cutaneous thermal injury, which can occur if tissue temperatures exceed 42–43 °C [27]. Patients with hypothermia have restricted cutaneous perfusion, causing applied heat to accumulate in superficial tissues rather than being absorbed and distributed by capillary circulation. This increases the risk of cutaneous thermal injury, which has been reported, particularly in cases involving improvised rewarming measures [28, 29]. Manufacturers of active external rewarming devices limit surface temperatures or discourage direct skin contact, recommending insulation between the skin and the heat source. Patients with intact cutaneous perfusion may tolerate higher temperatures without developing thermal injuries.

Patient comfort may be undervalued in mountain rescues. Results from our study show that providing active external warming results in increased mean skin temperatures (Fig. 3), which probably contribute to the documented increased thermal comfort alongside the increased esophageal temperature rewarming rate. The increased thermal comfort supports the use of active external rewarming. Feeling cold can heighten fear and discomfort [30], which is particularly relevant in mountain rescue operations that may last for several hours. Patients experiencing cold discomfort often experience pain from injury or other sources [31]. Providing external heat may enhance comfort, alleviate perceived pain, and potentially reduce analgesic requirements.

### Limitations

The direct validity of the rewarming rate observed in our study is limited to the described environment of cold air and does not necessarily reflect that of a clinical scenario, as the simulated accidental hypothermia involved healthy volunteers with a suppressed shivering response.

Although a washout period was used, and the order of the experiments were randomized, we cannot fully exclude potential period or learning effects inherent to the crossover design. Changing the protocol during the trial was necessary for safety reasons but may introduce a confounder in the design.

In the original trial used to develop the protocol for inhibition of shivering, additional Meperidine doses could be administered if oxygen consumption increased, indicating breakthrough shivering. We did not measure VO2 in our study, and therefore did not administer any supplemental doses. We shortened the interval from 30 to 25 min to avoid breakthrough shivering. We cannot guarantee that no breakthrough shivering occurred, but the crossover design reduces the influence on the results.

Subjective evaluations of comfort are susceptible to bias, as blinding of the intervention is impossible.

## Conclusion

The use of 3 active external rewarming devices resulted in a modest 0.2 °C/h increase in esophageal temperature during one hour of rewarming, compared to only using passive rewarming. The findings of this study suggest that nonshivering individuals may continue to experience a decline in esophageal temperature in a cold environment with passive rewarming alone. Active warming can help maintain or slightly increase the esophageal temperature.

## Supplementary Information

Supplementary Material 1.

Supplementary Material 2.

Supplementary Material 3.

Supplementary Material 4.

## Data Availability

The datasets used or analyzed in the current study are available from the corresponding author upon reasonable request.

## Abbreviations

ANCOVA: Analysis of covariance
ECG: Electrocardiogram
TBSA: Total body surface area

## Declarations

### Meetings

Preliminary results (not analyzed data) were presented at the annual congress of the International Commission for Alpine Rescue (ICAR) in Greece, October 2024.
