# Supplementary material for: Effect of active external rewarming on esophageal temperature in simulated prehospital accidental hypothermia: a randomized crossover trial
Source: Scand J Trauma Resusc Emerg Med. 2025 Dec 12;34:8. doi: 10.1186/s13049-025-01528-7 (PMC12805701; doi:10.1186/s13049-025-01528-7)
Supplement: Supplementary file 1 — Supplementary Material 1. [file 13049_2025_1528_MOESM1_ESM.docx]

**Inclusion criteria**

*Age: Over 18 years old*

*General health: Overall healthy*

*BMI: Below* 30 kg/m^2^

*Informed consent: Able to receive, understand and consent to partake in the experiment based on information provided about the experiment*

**Exclusion criteria**

*Allergies: Known allergy to Meperidine or Buspirone*

*Concomitant use of any of the following medications:* MAO-inhibitors, SSRI, Ritonavir, cimetidine, chlorpromazine, phenytoin, erythromycin, itraconazole, anti-platelet agents, Apomorphine.

*Pregnancy*

*Neurologic conditions:* Recent head trauma, high ICP or epilepsy

*General health:* Reduced liver- or kidney function, respiratory failure, cardiac arrhythmias or prolonged QTc, BPH, acute glaucoma, myasthenia gravis or previous cold injury/frost bite.
