# Supplementary material for: Effect of active external rewarming on esophageal temperature in simulated prehospital accidental hypothermia: a randomized crossover trial
Source: Scand J Trauma Resusc Emerg Med. 2025 Dec 12;34:8. doi: 10.1186/s13049-025-01528-7 (PMC12805701; doi:10.1186/s13049-025-01528-7)
Supplement: Supplementary file 2 — Supplementary Material 2. [file 13049_2025_1528_MOESM2_ESM.pdf]

MountainLab 2024

1

Part 1 - Before experiment

Research Participant ID ☐

Study day (A or B) ☐

Date (dd-mm-yy) ☐ - ☐ - ☐

Personal data

Age ☐ years

Sex ☐ male ☐ female

Pregnancy test result (if female) ☐ positive ☐ negative

Height ☐ cm

Weight ☐ kg

Checklist

Emergency bag ☐

Extra thermometer ☐

Informed consent

- Present ☐
- Signed ☐

Initial screening ☐

Self report form - General health ☐

MountainLab 2024

2

Part 2 - Before cooling

Research Participant ID	☐
Study day (A or B)	☐

Checkliste

No concurrent illness	☐	
Light breakfast	☐	
No tobacco last 24 h	☐	
Last 24 h		
No caffeine, alcohol or grapefruit	☐	
No hard physical exercise	☐	
Inclusion criteria achieved	☐ yes	☐ no
Exclusion criteria **not** achieved	☐ yes	☐ no

Tablet taken (hh:mm) ☐ : ☐

Checkliste

Corpuls	Attached	Turned on	Comments
Esophageal probe	☐	☐	
BP Cuff	☐	☐	
SpO2	☐	☐	
ECG	☐	☐	
Venflon	☐	☐	
Skin temperature sensors (7 pcs)	☐	☐	

Chamber sensors	Present
Temperature	☐
Humidity	☐

MountainLab 2024

3

Part 3 - Baseline

Research Participant ID ☐

Study day (A or B) ☐

Time (hh:mm) ☐ : ☐

Corpuls

- Esophageal temperature ☐ °C
- Heart rate ☐ /min
- BP Systolic ☐ mmHg
- BP Diastolic ☐ mmHg
- SpO2 ☐ %

Questionnarie

- Subjective shivering (0-2) ☐
- Objective shivering (0-3) ☐
- RASS (-5-4) ☐
- Cold discomfort (0-10) ☐
- Pain (0-10) ☐
- Nausea ☐ yes ☐ no
- If yes ☐ mild ☐ moderate ☐ severe
- Comments

MountainLab 2024

4a

Part 4a - During cooling - Subjective

Research participant ID [ ]

Study day (A or B) [ ]

Start cooling (hh:mm) [ ] : [ ]

	Klokkeslett (hh:mm)	Shivering subj (0-2)	Shivering obj (0-3)	RASS (-5 - 4)	Discomfort from cold (0-10)	pain (0-10)	Nausea yes	Nausea no	Nausea severity (1-3)
start 00:20	:								
00:40	:								
01:00	:								
01:20	:								
01:40	:								
02:00	:								

MountainLab 2024

4b

Part 4b - During cooling - Objective

Research Participant ID

Study day (A or B)

		Blood pressure					
		Standard			Safety measurement		
	Time (hh:mm)	sys	dia	<80? >200?	sys	dia	<80? >200?
start 00:20	:						
00:40	:						
01:00	:						
01:20	:						
01:40	:						
02:00	:						

MountainLab

5

Part 5 - Transition

Research participant ID [ ]

Study day (A or B) [ ]

Cooling stopped

Time (hh:mm) [ ] : [ ]

Temperature at termination [ ] °C

Cause
- [ ] Temperature (35°C)
- [ ] Time (2 h)
- [ ] Participant withdrawal
- [ ] Technical issue

Rewarming

Time start (hh:mm) [ ] : [ ]

Adverse events to report?

[ ] Yes [ ] No (If yes - AE form)

MountainLab

6

Part 6 - During rewarming

Research participant ID ☐

Study day (A or B) ☐

Subjective scores

	Time (hh:mm)		Shivering subj (0-2)	Shivering obj (0-3)	RASS (-5 - 4)	Discomfort from cold (0-10)	Pain (0-10)	Nausea yes	Nausea no	Nausea severity (1-3)
start 00:00		:								
00:20		:								
00:40		:								
01:00		:								

Objective scores

	Time (hh:mm)		Blood pressure Standard sys	Standard dia	Standard <80? >200?	Safety measurement sys	Safety measurement dia	Safety measurement <80? >200?
start 00:00		:						
00:20		:						
00:40		:						
01:00		:						

Adverse events to report?

☐ Yes ☐ No (If yes - AE form)

MountainLab 2024

7

Part 7 - After experiment

Research participant ID ☐

Study day (A or B) ☐

Checklist

Data transfer

- Corpuls ☐
- Sensor skin temperature ☐
- Ambient temperature sensor ☐
- Completed forms ☐

Adverse events to report?

☐ Yes ☐ No (If yes - AE form)
