# Supplementary material for: Effect of active external rewarming on esophageal temperature in simulated prehospital accidental hypothermia: a randomized crossover trial
Source: Scand J Trauma Resusc Emerg Med. 2025 Dec 12;34:8. doi: 10.1186/s13049-025-01528-7 (PMC12805701; doi:10.1186/s13049-025-01528-7)
Supplement: Supplementary file 3 — Supplementary Material 3. [file 13049_2025_1528_MOESM3_ESM.docx]

**Vurderes hvert 20. min:**

**SKJELVING**

Subjektiv vurdering: 0 = ingen skjelving, 1 = litt skjelving, 2 = skjelving.

Objektiv vurdering: BSAS:

| Score | Definisjon |
| --- | --- |
| 0 | Ingen skjelving |
| 1 | Mild skjelving lokalisert til nakke eller thorax |
| 2 | Moderat skjelving, utbredelse til øvre ekstremiteter |
| 3 | Voldsom skjelving, overkropp, øvre, og nedre ekstremiteter. |

**SEDASJON**

| Score | Beskrivelse |  |
| --- | --- | --- |
| +4 | Aggressiv | Åpenbart aggressiv eller voldlig. |
| +3 | Veldig agitert | Trekker ut tuber/ venfloner, aggressiv mot personale. |
| +2 | Agitert | Hyppig ikke-meningsfulle bevegelser movement, stritter i mot. |
| +1 | Urolig | Urolig eeller redd, ikke aggressive. |
| 0 | Våken og rolig | Gir spontant oppmerksomhet mot medarbeidere. |
| -1 | Trett | Ikke helt våken, med vedvarende (>10 sek) øyekontakt ved tiltale. |
| -2 | Lett sedert | Våkner kortvarig (<10 sek) med øyekontakt ved tiltale. |
| -3 | Moderat sedert | Bevegelse, men ingen øyekontakt ved tiltale. |
| -4 | Dypt sedert | Ingen respons ved tiltale, men bevegelse ved fysisk stimuli. |
| -5 | Ikke vekkbar | Ingen respons ved tiltale eller fysisk stimuli. |

**UBEHAG GRUNNET KULDE**

0 1 2 3 4 5 6 7 8 9 10

Ekstr. kald –svært kald – kald – kjølig – litt kjølig –nøytral – litt varm – varm – het – svært het – ekstr. het.

**SMERTE**

0 1 2 3 4 5 6 7 8 9 10

Ingen smerte Uutholdelig smerte

**KVALME**

JA/ NEI, hvis ja: mild, moderat eller kraftig.
