# Supplementary material for: Effect of active external rewarming on esophageal temperature in simulated prehospital accidental hypothermia: a randomized crossover trial
Source: Scand J Trauma Resusc Emerg Med. 2025 Dec 12;34:8. doi: 10.1186/s13049-025-01528-7 (PMC12805701; doi:10.1186/s13049-025-01528-7)
Supplement: Supplementary file 4 — Supplementary Material 4. [file 13049_2025_1528_MOESM4_ESM.docx]

**Assessment every 20. min:**

**Shivering**

Subjective assessment: 0 = no shivering, 1 = a little shivering, 2 = a lot of shivering.

Objective assessment: BSAS:

| Score | Definition |
| --- | --- |
| 0 | No shivering |
| 1 | Mild shivering neck/thorax |
| 2 | Moderate shivering, limited to upper body/extremities |
| 3 | Powerful shivering, torso and upper/lower extremities |

**Sedation (RASS)**


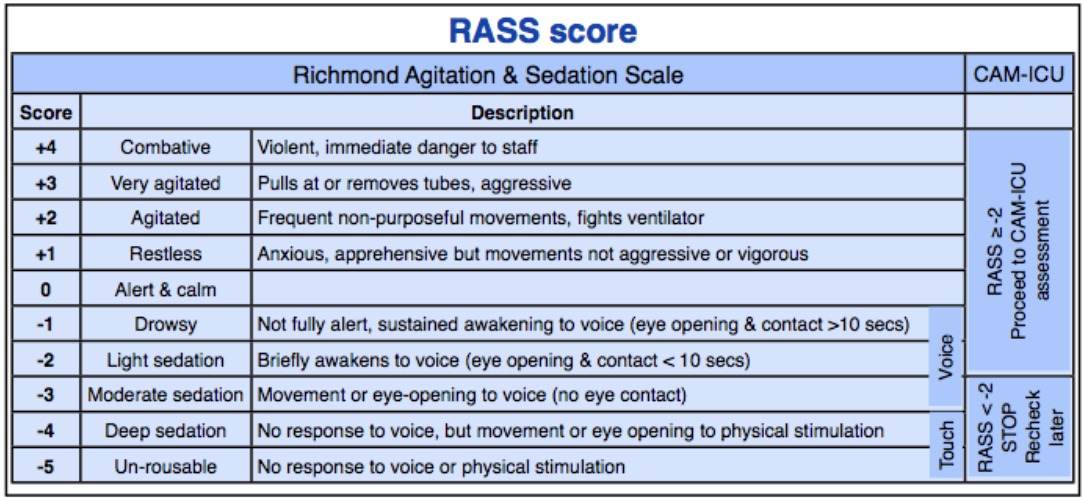


**Cold discomfort**

0 1 2 3 4 5 6 7 8 9 10

Extr. cold –very cold – cold – cool – slightly cool –neutral – slightly warm – warm – hot – very hot – extr. hot

**Pain**

0 1 2 3 4 5 6 7 8 9 10

No pain Excrutiating pain

**Nausea**

Yes/ No, if yes: mild, moderate or severe
